# Clinical Management Considerations for Craniopharyngioma in Geriatric Populations: An Assessment of the Surveillance, Epidemiology, and End Results Database

**DOI:** 10.3390/jcm15010195

**Published:** 2025-12-26

**Authors:** Jacob Gould, Arjit Singh, Saarang Patel, Noah Yaffe, Guan Li, Lou Blanpain, Julian Gendreau

**Affiliations:** 1 Kreiger School of Arts and Sciences, Johns Hopkins University, Baltimore, MD 21218, USA; 2 Department of Neuroscience, Johns Hopkins University, Baltimore, MD 21218, USA; asing152@jh.edu; 3 Department of Biological Sciences, Seton Hall University, South Orange, NJ 07079, USA; 4 Department of Neurological Surgery, Oregon Health & Science University, 3303 S. Bond Avenue, Building 1, Eighth Floor, Portland, OR 97239, USA; yaffe@ohsu.edu (N.Y.);; 5 Department of Neurological Surgery, University of California San Diego, San Diego, CA 92093, USA

**Keywords:** craniopharyngioma, elderly, resection, SEER, survival, very elderly

## Abstract

**Background**: Craniopharyngiomas are rare Sellar and suprasellar tumors that can cause significant visual, endocrine, and hypothalamic morbidity. Treatment decisions in older adults are complicated by higher comorbidity burden, reduced physiological reserve, and limited data especially for the very elderly. Because most studies group all patients ≥65 together, the survival impact of surgery and radiotherapy in elderly subgroups remains unclear. **Methods**: A retrospective cohort study was conducted using SEER 17 (2000–2022). Patients aged ≥50 years with histologically confirmed craniopharyngioma were categorized as non-elderly (50–64), elderly (65–79), or very elderly (≥80). Demographic and clinical characteristics were compared using Chi-square tests and two-sample *t*-tests. Overall survival (OS) was evaluated using Kaplan–Meier analyses and multivariable Cox proportional hazards models. **Results**: A total of 1259 patients met inclusion criteria. OS decreased with age. In the 50–64 cohort, subtotal resection (STR) and gross total resection (GTR) significantly improved survival, and radiotherapy provided additional benefit. In the 65–79 cohort, neither resection nor radiotherapy significantly influenced OS. In the ≥80 cohort, STR was associated with worse survival relative to no resection (HR 2.21; *p* = 0.0349), while GTR and radiotherapy did not improve outcomes. **Conclusions**: The effect of craniopharyngioma treatment varies by age. Surgery and radiotherapy benefit younger patients, whereas adults aged 65–79 may gain symptomatic but not survival advantages. In patients ≥80, STR may reduce survival, underscoring the need for individualized treatment plans aligned with patient goals and physiological reserve.

## 1. Introduction

Craniopharyngiomas are rare epithelial tumors arising from remnants of Rathke’s pouch within the Sellar and suprasellar region, accounting for approximately 1–4% of all primary intracranial neoplasms [1]. Despite their benign histology, these tumors frequently exert mass effect on adjacent critical neurovascular and endocrine structures which can cause substantial morbidity related to hypothalamic dysfunction and lifelong endocrinopathies [2]. Management typically includes a combination of surgical resection and radiotherapy with the goal of balancing durable tumor control and symptom management against the risk of treatment-related complications [3].

Optimal management of craniopharyngioma in elderly populations remains poorly defined, and even less well defined in very elderly cohorts (age 80+ years). Elderly patients aged 65 years and older represent a rapidly expanding demographic with high baseline comorbidity burden, reduced physiological reserve, and unique considerations surrounding operative tolerance and postoperative recovery. Although age is consistently associated with greater perioperative risk and diminished long-term resilience, it is uncertain how these factors interact with craniopharyngioma biology, tumor behavior, and the potential benefits of intervention in this elderly age cohort [4,5]. Existing population-based studies typically categorize “elderly” patients as those aged ≥65 or ≥70 years, but this broad grouping may obscure biologically and clinically meaningful differences among different elderly cohorts. As a result, clinicians can face substantial uncertainty when determining whether surgical resection, radiotherapy, or conservative management offers meaningful survival benefits for elderly individuals and very elderly individuals [6].

To address this knowledge gap, we analyzed patients aged ≥65 years with craniopharyngioma in the Surveillance, Epidemiology, and End Results (SEER) database. We stratified the elderly population into two groups: elderly (ages 65–79) and very elderly (80+) to resolve potential differences between the elderly and the very elderly. We sought to identify demographic and treatment-related factors associated with overall survival (OS) in these cohorts and to evaluate whether surgical or radiation-based interventions influence measurable survival outcomes. By characterizing outcomes within this less frequently studied population, our goal is to inform clinical decision-making and contribute to evidence-based management strategies for elderly and very elderly diagnosed with craniopharyngioma.

## 2. Methods

### 2.1. Database

We conducted a retrospective cohort assessment using the SEER 17 database (2000–2022), a de-identified, publicly accessible dataset maintained by the National Cancer Institute. SEER compiles population-based cancer incidence and survival information from 17 registries that collectively cover approximately 26.5% of the U.S. population. These registries draw data from hospitals and outpatient practices across multiple geographic regions, including the West, Midwest, Southwest, Northeast, and Southeast [7]. Because all SEER records are anonymized and freely available for public use, institutional review board oversight and individual patient consent were not required for this study.

### 2.2. Case Selection

Patients were identified through the SEER topography code C70.0 (cerebral meninges), C71.0 (cerebrum), C71.1 (frontal lobe), C71.5 (ventricle, NOS), C71.7 (brain stem), C71.8 (overlapping lesion), C71.9 (brain, NOS), C72.5 (cranial nerve, NOS), C72.9 (nervous system, NOS), C75.1 (pituitary gland), C75.2 (craniopharyngeal duct region). Cases meeting ICD-O-3 histologic criteria for craniopharyngioma (9350/0, 9350/1, 9350/3, 9351/0, 9351/1, 9351/3, 9352/1) were included. To ensure uniform reporting of operative variables, we restricted the cohort to pathologically confirmed diagnoses from 2000 onward, when SEER began systematically documenting extent of surgical resection [7].

Individuals were eligible if they were 50 years of age or older at diagnosis, after which they were categorized into a non-elderly comparison group (50–64 years), an elderly group (65–79 years), and a very elderly group (≥80 years). The very elderly and elderly cohorts were the cohorts of interest, while the non-elderly cohort was included for purposes of comparison. Patients were required to have complete data for age, survival time, survival status, sex, race, ethnicity, median household income, marital status, geographic residence, extent of resection, and tumor size. Those younger than 50 years or with missing or unknown values for any required variable were excluded. The age groupings for the elderly and very elderly were based on existing literature. Geriatric populations are often grouped into three categories: the youngest-old (65–74), the middle old (75–84) and the oldest old (85+) [8]. To align these to a two group classification (elderly vs. very elderly), the midpoint of the existing classifications was taken to identify elderly as 65–79 and very elderly as 80+. This classification creates a distinction between geriatric populations to acknowledge differences in their distinct physiological vulnerabilities and operative risk profiles [9]. Although our age stratification was informed by widely used geriatric categorizations in neurosurgical and oncologic research, these thresholds may not fully capture craniopharyngioma-specific operative risk or tumor biology. Chronological age likely serves as an imperfect surrogate for physiological reserve, frailty, and comorbidity burden, which may vary substantially among individuals within the same age category. Accordingly, our findings should be interpreted as age-associated population-level trends rather than deterministic treatment thresholds.

### 2.3. Surgical Classification

Extent of resection was determined using SEER surgery codes, which were reviewed in accordance with SEER coding guidelines to maintain consistency across reporting years. For tumors classified under topography codes C75.1 and C75.2, gross total resection (GTR) was defined by surgery codes 30 and 50, representing amputation or major amputation procedures consistent with complete tumor removal. Subtotal resection (STR) corresponded to codes 20 and 30, which include local excision, partial resection, or radical excision that does not meet criteria for major amputation. For tumors classified under topography codes C70.0–72.9, GTR was defined by surgery code 55 and STR corresponded to codes 10, 20, and 40. Patients assigned code 00 were considered to have undergone no resection, while those coded within the 90-series (indicating unspecified or unknown surgery) were excluded from the assessment. In instances where operative data and pathology data differed, the pathology data was used as the definitive source for determining extent of resection.

### 2.4. Patient, Tumor, and Treatment Characteristics

Variables extracted from the SEER dataset included age at diagnosis, sex, race (White, Black, Asian/Pacific Islander, or Other), ethnicity (Hispanic vs. non-Hispanic), household income, marital status, geographic residence, survival time, and survival status. Treatment-related data consisted of the extent of surgical resection. Additional tumor-specific attributes including tumor size, histologic grade, margin status, and coexisting malignancies were not incorporated into the multivariable assessment because these fields are reported inconsistently for craniopharyngiomas in SEER. Comparisons between the non-elderly, elderly, and very elderly groups were conducted using Chi-square tests for categorical variables and two-sample *t*-tests for continuous variables.

### 2.5. Outcome Measures and Statistical Assessment

The primary study endpoint was overall survival (OS), defined as the number of months from diagnosis to death or last available follow-up. Because SEER does not provide craniopharyngioma specific mortality data, disease-specific survival could not be evaluated, and OS was therefore selected as the principal outcome measure. Patients who were alive at their most recent follow-up were censored at that time point. Kaplan–Meier curves were generated to compare survival across treatment categories within each cohort, and differences between curves were tested using the log-rank method.

Separate multivariable Cox proportional hazards models were constructed for individuals aged in each cohort. Propensity score matching and inverse probability weighting were considered; however, the small sample size of the ≥80-year population did not permit stable matched or weighted analyses. Covariates incorporated into the Cox models included sex, race, ethnicity, household income, marital status, geographic residence, extent of resection (none, STR, GTR), and tumor size.

Hazard ratios (HRs) with corresponding 95% confidence intervals were reported. Statistical significance was defined as *p* < 0.05. Assumptions of proportional hazards were evaluated using Schoenfeld residuals and were found to be satisfied. Missing data was addressed through listwise deletion because imputation was not appropriate for categorical registry variables. All statistical analyses were performed using R version 4.5.1 (R Core Development Team). Artificial intelligence was not used in the production of this manuscript beyond ensuring proper formatting of citations in adherence with formatting of the American Medical Association. The study adhered to the STROBE guidelines for reporting observational research [10].

## 3. Results

### 3.1. Patient Clinical Characteristics and Demographics

After application of inclusion criteria outlined above, a total of 1259 patients aged ≥50 years were included in the assessment (Table 1). The mean age at diagnosis of craniopharyngioma, by definition and nature of the study structure, differed significantly across cohorts 56.90 ± 4.29 (50–64 years), 70.73 ± 4.15 (65–79 years) and 83.69 ± 3.02 years (≥80 years); *p* < 0.001). The overall sex distribution was roughly balanced though the fraction of afflicted males declined significantly with advancing age with 51.2% male in 50–64 years, 47.7% male in 60–79 years, and 33.8% male in ≥80 years; *p* = 0.013).

Racial composition was predominantly White across all age groups (68.5%, 72.0%, 79.7%) in the 50–64 years, 60–79 years, and ≥80 years cohorts, respectively. The next most predominant race was Black, followed by Asian/Pacific Islander patients. Racial composition did not differ significantly between groups (*p* = 0.337). Most patients were non-Hispanic, and such status did not differ between age groups. Median household income and residence type did not differ significantly between age groups with most patients residing in urban areas and reporting annual incomes ≥ $70,000. Marital status differed significantly with age (*p* < 0.001). The proportion of married patients decreased from 62.0% to 61.8% to 39.2% in the 50–64 years, 60–79 years, and ≥80 years cohorts respectively. The corresponding widowed status increased in these respective age groups from 5.1% to 11.3% to 43.2%.

Extent of surgical resection differed significantly across cohorts (*p* < 0.001). The proportion of patients not undergoing resection increased significantly with age remained high (16.6% (50–64 years), 28.4% (65–79 years) and 58.1% (≥80 years); *p* < 0.001), while GTR rates declined slightly with age (14.9% (50–64 years), 10.4% (65–79 years) and 9.5% (≥80 years); *p* < 0.001). Rates of chemotherapy administration were near 0% across cohorts and did not differ significantly between cohorts, while radiation therapy usage decreased significantly with age (20.9% (50–64 years), 16.1% (65–79 years) and 9.5% (≥80 years); *p* = 0.014).

### 3.2. Survival Assessment

Mean and median overall survival (OS) declined progressively with increasing age. Among patients aged 80 years and older, mean OS was 42.7 months with no resection, 26.1 months with subtotal resection (STR), and 67.9 months with gross total resection (GTR). In comparison, patients aged 65–79 years demonstrated mean survivals of 56.5, 53.5, and 71.4 months following no resection, STR, and GTR, respectively. The 50–64 cohort exhibited the longest OS across all resection strategies (mean OS: 60.8, 73.2, and 105.0 months, respectively).

**Figure 1:**
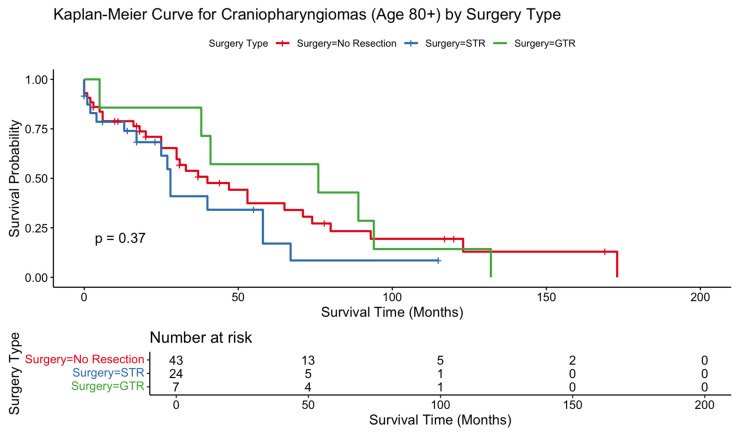
Kaplan–Meier curve depicting overall survival difference between patients 80 or older who received gross total resection vs. subtotal resection vs. no surgical procedure for craniopharyngioma.

**Figure 2:**
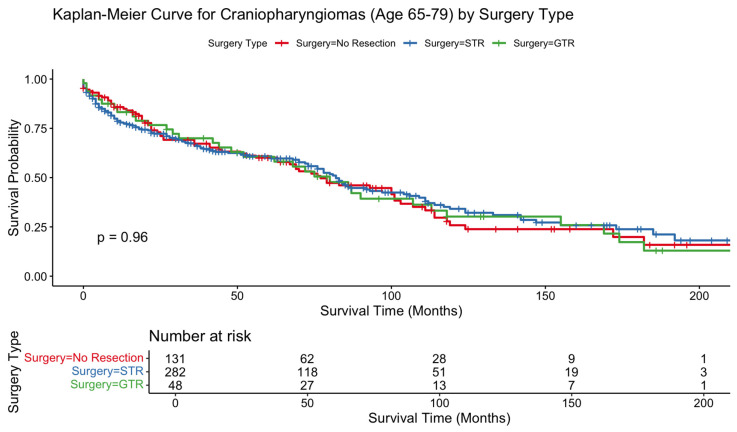
Kaplan–Meier curve depicting overall survival difference between patients aged 65–79 who received gross total resection vs. subtotal resection vs. no surgical procedure for craniopharyngioma.

**Figure 3:**
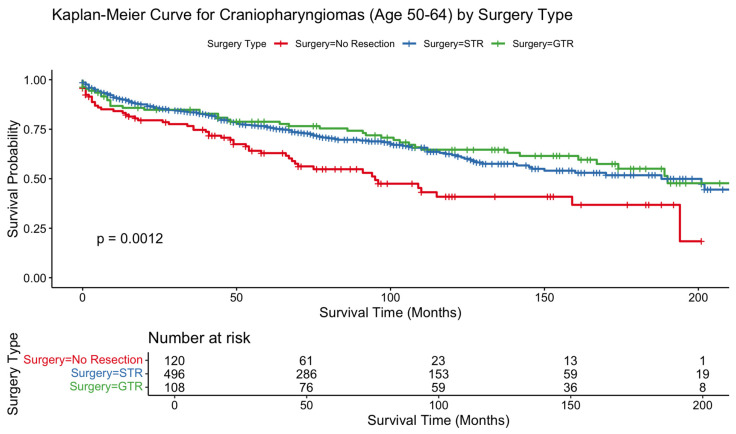
Kaplan–Meier curve depicting overall survival difference between patients aged 50–64 who received gross total resection vs. subtotal resection vs. no surgical procedure for craniopharyngioma.

Kaplan–Meier analyses showed no statistically significant differences in survival across resection types in the 80+ cohort (*p* = 0.37) or the 65–79 cohort (*p* = 0.96). In contrast, patients aged 50–64 demonstrated a significant OS difference by surgical extent, favoring resection (*p* = 0.0012). In patients 80+, survival curves began with substantial overlap but diverged gradually over time, with STR exhibiting the lowest long-term survival; however, this separation did not reach significance. The 65–79 survival curves were overlapping across treatment groups, consistent with nonsignificant Cox model findings. In contrast, the 50–64 cohort showed clear and sustained survival benefit for surgically treated patients over the entire follow-up interval (Figure 1, Figure 2 and Figure 3).

In the 80+ cohort, STR was associated with significantly worse survival relative to no resection (HR 2.21, 95% CI 1.06–4.61; *p* = 0.0349). GTR did not reach statistical significance (HR 1.07, *p* = 0.89). No other demographic or treatment-related covariates, including sex, race, ethnicity, income, marital status, residence, or radiotherapy, demonstrated significant associations with OS in this age group. In the 65–79 cohort, neither STR nor GTR significantly influenced OS, consistent with the overlapping Kaplan–Meier curves. Female sex remained independently protective (HR 0.66, *p* = 0.0023), whereas Black race was associated with higher mortality (HR 1.76, *p* = 0.0012). Radiotherapy also did not significantly affect OS in this cohort. In the 50–64 cohort, both STR (HR 0.71; *p* = 0.032) and GTR (HR 0.60; *p* = 0.017) were associated with improved survival. Higher income and married marital status were also protective. In contrast to older cohorts, radiation therapy significantly improved OS in this group (HR 0.70, 95% CI 0.50–0.97; *p* = 0.0347). Black race remained a predictor of increased mortality (HR 1.61, *p* = 0.0015). Results are summarized in Table 2.

## 4. Discussion

In this population-based study of elderly and very elderly patients with craniopharyngioma, we observed age-stratified differences in the association between surgical intervention, radiotherapy, and overall survival. These findings support a nuanced, age-sensitive approach to management that aligns treatment intensity with patient-specific goals, physiological reserve, and expected clinical benefit.

Among patients younger than 65, both STR and GTR were associated with substantial improvements in survival, and radiotherapy independently conferred a significant protective effect. These results are consistent with prior literature demonstrating the survival advantage of resection and adjuvant therapy in younger, medically fit individuals, for whom operative risk is lower and long-term tumor control is a primary goal [11]. In these patients, aggressive management, including maximal safe resection followed by radiotherapy when indicated, remains a central component of standard care. Similar to prior brain tumor studies, we also observed, in this cohort, that Black race was associated with worse outcomes, consistent with well-documented disparities in neuro-oncologic care access, comorbidity burden, and treatment timeliness [12].

In contrast, patients aged 65–79 years did not exhibit significant survival benefit from either STR or GTR. Radiotherapy similarly did not influence survival in this cohort. While surgical intervention did not confer significant benefit to OS, it also did not confer significant harm to OS. These observations align with geriatric neurosurgical literature showing that older adults experience greater perioperative risk yet may still achieve symptom relief, visual improvement, and endocrine stabilization following surgical intervention [13]. Given the morbidity often associated with craniopharyngioma, including visual decline, obstructive hydrocephalus, and progressive hypothalamic dysfunction, surgical or radiotherapeutic treatment may still provide meaningful clinical benefit even in the absence of measurable survival prolongation. Thus, for adults aged 65–79, decisions regarding resection should be individualized, balancing tumor-related symptoms, comorbidities, and patient preferences rather than survival metrics alone. In this cohort, female sex was associated with improved survival, a finding consistent with prior literature on other intracranial tumors thought to relate to both hormonal and non-hormonal biologic differences in tumor behavior and systemic resilience [14].

The 80+ cohort demonstrated a distinct pattern: STR was independently associated with worse survival relative to no resection, and neither GTR nor radiotherapy conferred benefit. Kaplan–Meier curves further illustrated a late separation in survival favoring conservative management or GTR over STR, even though differences did not reach statistical significance. These findings underscore the unique vulnerability of the very elderly, in whom diminished physiological reserve, frailty, and comorbidity burden may outweigh potential advantages of surgical debulking. Similar trends have been reported in studies of very elderly patients undergoing intracranial tumor resections, where operative morbidity and postoperative decline substantially impact mortality and quality of life [5,15,16]. Importantly, the association between subtotal resection and worse survival in patients aged 80 years and older should not be interpreted as evidence that STR itself is causally harmful. Rather, this finding likely reflects substantial selection and indication bias. Subtotal resection is often pursued in the setting of larger, more symptomatic, or surgically urgent tumors, including those causing visual compromise or hydrocephalus, in patients who may already be physiologically vulnerable. Additionally, very elderly patients selected for STR may have had greater baseline frailty or comorbidity burden that is not captured in SEER. As such, the observed survival decrement likely reflects underlying disease severity and patient vulnerability rather than the surgical strategy alone. Thus, in patients aged 80 and older, the role of surgery should be weighed carefully against patient goals and quality of life expectations. Considerations around symptom relief should be balanced around potential diminished OS with clear communication about potential postoperative survival outcomes.

It is important to note that radiotherapy utilization declined substantially with advancing age, which likely reflects a combination of medical comorbidity, concerns regarding tolerance, treatment intent (palliative vs. definitive), and patient or provider preference. This underutilization may obscure potential survival associations in older cohorts, particularly if radiotherapy was selectively withheld from patients perceived to have limited life expectancy or frailty. As SEER does not capture treatment intent or performance status, the observed age-dependent effects of radiotherapy should be interpreted cautiously.

Collectively, these age-stratified findings highlight that chronological age alone does not dictate the appropriateness of intervention. For elderly and very elderly patients, overall survival is often secondary to goals such as symptom relief, visual preservation, avoidance of acute neurologic deterioration, and maintenance of independence. Surgical or radiotherapeutic intervention may therefore provide meaningful clinical benefit even in the absence of measurable survival prolongation. This data reinforces current recommendations that management of craniopharyngioma in the elderly population should be individualized and guided by patient-specific goals, anticipated quality-of-life outcomes, physiological reserve, and the likelihood of treatment-related morbidity [17].

## 5. Limitations

This study carries several limitations inherent to retrospective registry-based analyses. SEER does not capture key clinical variables such as functional tumor status, hormonal subtype, comorbidities, perioperative complications, or treatment intent (curative vs. palliative), all of which may influence overall survival. Specifically, several key clinical variables central to craniopharyngioma decision-making are not captured within SEER, including tumor subtype (adamantinomatous vs. papillary), degree of hypothalamic involvement, baseline endocrine dysfunction, cystic versus solid composition, symptom burden prompting intervention, and validated comorbidity or frailty indices. These omissions introduce unavoidable residual confounding. In particular, hypothalamic involvement (one of the strongest predictors of long-term morbidity and mortality in craniopharyngioma) cannot be accounted for and may substantially influence both treatment selection and outcomes independent of extent of resection. Selection bias is also possible, as patients chosen for surgery are typically healthier than those managed conservatively. Additionally, the number of patients aged ≥80 years was relatively small, limiting power to detect subtle associations in this subgroup. Although our multivariable models identified significant predictors, unmeasured confounders may still explain part of the observed relationships. For example, tumor volume size may have predictive effect on survival, but such data was not incorporated in this study due to heterogeneity and lack of data availability within SEER. Future work employing propensity-based methods and validating these findings in external clinical datasets will be essential for reducing residual bias.

Our analysis was restricted to overall survival and did not assess postoperative functional outcomes or quality of life. Thus, while STR was associated with reduced survival in the very elderly and improved survival in younger patients, its impact on symptom relief, return to baseline function, and long-term quality of life were not measured though should be considered carefully with the patient in clinical decision-making. Furthermore, some surgeries, particularly in older patients, may have been undertaken primarily for symptom control rather than for prolonging life. Because SEER does not systematically record treatment intent, these nuances may partially confound survival estimates. Prospective, multicenter studies that integrate physiological metrics, tumor-specific molecular data, patient-reported outcomes, and explicit documentation of therapeutic intent are needed to refine prognostic frameworks for this diverse patient population.

## 6. Conclusions

Craniopharyngioma management in older adults requires an age-sensitive and individualized approach. In patients younger than 65, both surgical resection and radiotherapy were associated with meaningful improvements in overall survival. Among adults aged 65–79, treatment did not significantly alter survival but may still provide important symptom relief without compromising longevity. In contrast, subtotal resection in patients aged 80 and older was associated with decreased survival, underscoring the need for cautious surgical selection and alignment of treatment plans with patient goals, physiological reserve, and anticipated quality of life. These findings highlight the heterogeneity within elderly populations and support tailoring intervention strategies to optimize outcomes across the aging spectrum.

## Figures and Tables

**Table 1:** Demographics of Craniopharyngioma Patients Stratified by Very Elderly (Aged 80+ years), Elderly (Aged 65–79 yr), and Elderly (Aged 50–64 yr) (N = 1259).

	Demographics			
	Elderly Cohort ≥80 Years Old (N = 74)	Elderly Cohort 65–79 Years Old (N = 461)	Elderly Cohort 50–64 Years Old (N = 724)	*p*-Value
**Age**	83.69 (3.02)	70.73 (4.15)	56.90 (4.24)	**<0.001**
**Sex**				**0.013**
Male	25 (33.8%)	220 (47.7%)	371 (51.2%)	
Female	49 (66.2%)	241 (52.3%)	353 (48.8%)	
**Race**				0.337
White	59 (79.7%)	332 (72.0%)	496 (68.5%)	
Black	8 (10.8%)	78 (16.9%)	149 (20.6%)	
Native American	1 (1.4%)	3 (0.7%)	6 (0.8%)	
Asian/Pacific Islander	6 (8.1%)	48 (10.4%)	73 (10.1%)	
**Ethnicity**				0.648
Not Hispanic	66 (89.2%)	401 (87.0%)	621 (85.8%)	
Hispanic	8 (10.8%)	60 (13.0%)	103 (14.2%)	
**Median Household Income**				0.552
<70,000 k/year	14 (18.9%)	114 (24.7%)	175 (24.2%)	
≥$70,000 k/year	60 (81.1%)	347 (75.3%)	549 (75.8%)	
**Marital Status**				**<0.001**
Single	6 (8.1%)	70 (15.2%)	147 (20.3%)	
Married	29 (39.2%)	285 (61.8%)	449 (62.0%)	
Divorced	7 (9.5%)	54 (11.7%)	85 (11.7%)	
Widowed	32 (43.2%)	52 (11.3%)	43 (5.9%)	
**Residence**				0.211
Rural	13 (17.6%)	50 (10.8%)	79 (10.9%)	
Urban	61 (82.4%)	411 (89.2%)	645 (89.1%)	
**Extent of Resection**				**<0.001**
No Resection	43 (58.1%)	131 (28.4%)	120 (16.6%)	
Subtotal Resection	24 (32.4%)	282 (61.2%)	496 (68.5%)	
Gross Total Resection	7 (9.5%)	48 (10.4%)	108 (14.9%)	
**Chemotherapy**				0.854
Not Performed	74 (100.0%)	459 (99.6%)	721 (99.6%)	
Performed	0 (0.0%)	2 (0.4%)	3 (0.4%)	
**Radiotherapy**				**0.014**
Not Performed	67 (90.5%)	387 (83.9%)	573 (79.1%)	
Performed	7 (9.5%)	74 (16.1%)	151 (20.9%)	

**Table 2:** Multivariable Cox Proportional Hazards Models of Overall Survival for patients with craniopharyngioma stratified by age.

Cox Proportional Hazards Model	Elderly Cohort ≥80 Years Old (N = 74)		Elderly Cohort 65–79 Years Old (N = 461)		Elderly Cohort 50–64 Years Old (N = 724)	
	Hazard Ratio [95% CI]	*p*-Value	Hazard Ratio [95% CI]	*p*-Value	Hazard Ratio [95% CI]	*p*-Value
**Sex**						
Male	Ref.		Ref.		Ref.	
Female	0.494 [0.170, 1.434]	0.195	0.664 [0.510, 0.864]	**0.002**	0.896 [0.696, 1.151]	0.388
**Race**						
White	Ref.		Ref.		Ref.	
Black	2.330 [0.823, 6.592]	0.111	1.756 [1.249, 2.469]	**0.001**	1.613 [1.200, 2.167]	**0.002**
Native American	N/A	N/A	1.368 [0.336, 5.574]	0.662	1.957 [0.709, 5.401]	0.194
Asian/Pacific Islander	0.376 [0.084, 1.673]	0.199	0.849 [0.506, 1.424]	0.536	0.715 [0.421, 1.212]	0.213
**Ethnicity**						
Not Hispanic	Ref.		Ref.		Ref.	
Hispanic	2.284 [0.849, 6.146]	0.102	1.407 [0.963, 2.057]	0.078	0.888 [0.582, 1.356]	0.582
**Median Household Income**						
<70,000 k/year	Ref.		Ref.		Ref.	
≥$70,000 k/year	0.312 [0.087, 1.123]	0.075	0.769 [0.556, 1.062]	0.111	0.540 [0.397, 0.734]	**<0.001**
**Marital Status**						
Single	Ref.		Ref.		Ref.	
Married	0.328 [0.078, 1.374]	0.127	0.704 [0.491, 1.009]	0.056	0.533 [0.391, 0.727]	**<0.001**
Divorced	0.604 [0.123, 2.962]	0.534	0.825 [0.521, 1.305]	0.412	1.028 [0.686, 1.541]	0.894
Widowed	0.343 [0.090, 1.307]	0.117	0.989 [0.628, 1.557]	0.962	0.685 [0.396, 1.183]	0.174
**Residence**						
Rural	Ref.		Ref.		Ref.	
Urban	1.378 [0.347, 5.480]	0.649	0.794 [0.502, 1.257]	0.326	1.503 [0.960, 2.353]	0.074
**Extent of Resection**						
No Resection	Ref.		Ref.		Ref.	
Subtotal Resection	2.207 [1.058, 4.605]	**0.035**	1.072 [0.802, 1.434]	0.637	0.705 [0.512, 0.971]	**0.032**
Gross Total Resection	1.069 [0.419, 2.722]	0.891	0.928 [0.608, 1.416]	0.727	0.603 [0.398, 0.913]	**0.017**
**Radiotherapy**						
Not Performed	Ref.		Ref.		Ref.	
Performed	1.252 [0.464, 3.383]	0.657	1.033 [0.733, 1.454]	0.854	0.700 [0.503, 0.975]	**0.035**

## Data Availability

The data that supports the findings of this study are publicly available from the Surveillance, Epidemiology, and End Results (SEER) Program of the National Cancer Institute. Access to SEER data and code can be obtained upon request to the authors, and raw data can be found at: https://seer.cancer.gov/data-software/ (accessed on 10 November 2025).
